# Malaria epidemiology in Suriname from 2000 to 2016: trends, opportunities and challenges for elimination

**DOI:** 10.1186/s12936-018-2570-4

**Published:** 2018-11-12

**Authors:** Hélène Hiwat, Beatriz Martínez-López, Hedley Cairo, Loretta Hardjopawiro, Agatha Boerleider, Elisabeth Carmen Duarte, Zaida E. Yadon

**Affiliations:** 1 Ministry of Health Malaria Programme, Ministry of Health, Paramaribo, Suriname; 2 Center for Animal Disease Modeling and Surveillance (CADMS), Department of Medicine & Epidemiology, School Veterinary Medicine, University of California, Davis, CA USA; 3 Faculty of Medicine, Universidade de Brasília (UnB), Brasília, DF Brazil; 4 Communicable Diseases Health Analysis, Pan American Health Organization, Washington, DC USA

**Keywords:** Malaria programme, Malaria elimination, *Plasmodium falciparum*, *Plasmodium vivax*, Space–time cluster analysis, Regional collaboration

## Abstract

**Background:**

Suriname has experienced a significant change in malaria transmission risk and incidence over the past years. The country is now moving toward malaria elimination. The first objective of this study is to describe malaria epidemiological trends in Suriname between 2000 and 2016. The second objective is to identify spatiotemporal malaria trends in notification points between 2007 and 2016.

**Methods:**

National malaria surveillance data resulting from active and passive screening between 2000 and 2016 were used for the temporal trend analysis. A space–time cluster analysis using SaTScan™ was conducted on Malaria Programme-data from 2007 to 2016 comparing cases (people tested positive) with controls (people tested negative).

**Results:**

Suriname experienced a period of high malaria incidence during 2000–2005, followed by a steep decline in number of malaria cases from 2005 onwards. Imported malaria cases, mostly of Brazilian nationality and travelling from French Guiana, were major contributors to the reported number of cases, exceeding the national malaria burden (94.2% of the total). Most clusters in notification points are found in the border area between Suriname and French Guiana. Clustering was also found in the migrant clinic in Paramaribo.

**Conclusions:**

Suriname has successfully reduced malaria to near-elimination level in the last 17 years. However, the high malaria import rate resulting from cross-border moving migrants is a major challenge for reaching elimination. This requires continued investment in the national health system, with a focus on border screening and migrant health. A regional approach to malaria elimination within the Guianas and Brazil is urgently needed.

## Background

An estimated 216 million cases of malaria occurred worldwide in 2016 [1]. Malaria incidence and mortality have been decreasing on a global scale between 2000 and 2015. More than half of the malaria-endemic countries achieved reductions in new malaria cases of at least 75% [2]. Malaria-endemic countries on the American continent, having experienced significant decreases in number of cases, contribute only a fraction to the global malaria burden. Several of these countries are moving towards elimination [3, 4]. Suriname is part of the Guianas (Suriname, Guyana, French Guiana) and had the highest annual parasite incidence (API) and concentration of *Plasmodium falciparum* cases in the Americas in 2004 [3, 5]. It has reported a significant decline in the number of cases since then after successful implementation of prevention and control interventions [6–8]. Suriname is committed to the goal of eliminating malaria by 2020 [9] but faces important challenges, including the reception of imported cases from other endemic countries in the region [6], and ongoing malaria transmission in remote gold mining areas [10].

In 2015, the World Health Organization launched a Global Technical Strategy for malaria 2016–2030 [11]. The strategy focuses on improved access to diagnosis and treatment and turning malaria surveillance into a core intervention. Malaria surveillance has become essential to identify high-risk areas for malaria and to guide the implementation of risk-based prevention and control strategies in the American region [11–14]. This study aims to describe the malaria morbidity and mortality trends and the geographical distribution of malaria in Suriname, taking into account the most probable origin of infection, for the period 2000 to 2016. Secondly, it aims to assess the spatiotemporal trends of malaria diagnosed in the health system notification points using data from the Suriname surveillance programme from 2007 to 2016. The overall objective is to evaluate the evolution of the disease in the last 17 years as well as to identify challenges and guide future interventions in order to achieve elimination in the country. The space–time cluster analysis of notification points will help identify priority service points, and allow for determination of risk population characteristics in these service points. This in turn enables better-targeted investments and interventions.

## Methods

### Study design

This is a descriptive study using routinely collected malaria surveillance data. First, the temporal evolution of national malaria cases from 2000 to 2016 was evaluated. Secondly, a space–time cluster-analysis using a Bernoulli model was conducted for the sub-set of information gathered during the 2007–2016 period, which is when both high resolution geographical and temporal data were available.

### Study setting

Suriname is a malaria-endemic country along the northern coast of South America. The coastal area has been free of malaria since 1968; however, the interior has recorded a high malaria incidence and prevalence in the early years of this Millennium (around 160 malaria cases/1000 persons at risk per year). Since then, malaria incidence decreased sharply and steadily to elimination level from 2004 to 2009 [1, 2], with sporadic outbreaks in gold-mining areas.

The tropical rainforests in the interior provide excellent habitat to the main malaria vector *Anopheles darlingi* [15]. Secondary (potential) vectors include *Anopheles nuneztovari* and *Anopheles oswaldoi* [16]. The mass distribution of long-lasting impregnated bed nets between 2006 and 2009 and repeated large river flooding in 2006 in high-transmission risk areas are thought to have negatively impacted the *An. darlingi* populations in these areas during that time [17]. The interior, however, continues to be an important risk area, since this vector has proven efficient in malaria transmission even in low densities [15].

Road infrastructure in the interior of the country is very limited. Priority modes of transportation are boats (dug-out canoes) and small airplanes, as well as all-terrain vehicles in remote mining areas, resulting in challenging logistics for the provision of health services.

Malaria parasite species identified in Suriname include *P. falciparum*, *Plasmodium vivax* and *Plasmodium malariae.* Mixed infections have been reported.

### Study population

The study population consisted of all subjects who were tested for malaria, and which are recorded in the Surinamese national surveillance database. The population at risk in Suriname is composed of stable and mobile populations in the interior of the country. The stable populations are Maroon (descendants of African slaves) and Amerindian (native) populations living in tribal villages along rivers in the forests of the interior. Both the Maroon and Amerindian populations consist of several tribes, each with its own language. Being of African descent, many of the Maroons have a Duffy-negative phenotype which prevents them from becoming infected by *P. vivax.*

Since 2007, the population at risk was extended to include the mobile gold-mining communities in remote areas in the forest. These are mostly migrant miners of Brazilian origin (Portuguese speaking), but also include a small portion of Surinamese Maroons [18], Chinese and nationals of regional (Latin-American) countries. The total number of population at risk varied from 47,372 in 2000 to 84,700 in 2016. This increase was due to both stable population growth and the inclusion of mobile migrants. The number of Maroons and Amerindians are based on health registration data, since most people in the villages are registered at the Medical Mission Primary Health Care organization (Medical Mission) since birth. The Medical Mission is a government-funded, non-governmental organization providing primary health care to the stable communities in the interior. The number of mobile migrants is unknown and varies depending, among other things, on gold availability, gold price and military counter-intervention in neighbouring countries (especially in French Guiana). It is estimated at 20,000 people.

### Malaria interventions

The most important malaria interventions during the study period included: (i) passive screening, and health education at central level and in the villages of the interior; (ii) bed net production and distribution in cooperation with local women organizations until 2006; (iii) introduction of artemisinin-combination therapy (ACT) for *P. falciparum* infections at the end of 2004; (iv) mass distribution of free long-lasting insecticide-treated nets ((LLINs) and retreatment-tablets) in combination with a large awareness campaign between 2006 and 2009, followed by regular distribution of free LLINs at screening points and during active case detection surveys (ACDs) in high-risk areas since then; (v) indoor residual spraying (IRS) in high-risk areas in 2006 (discontinued following a steep decrease in number of cases); (vi) national introduction of rapid diagnostic tests (RDTs) in 2006; (vii) introduction of single-dose primaquine in addition to ACT for *P. falciparum* infections in 2007; and, (viii) passive and active case detection surveys (ACDs) in remote risk areas (mining areas) since 2009. Implementation of (changes of) nationwide interventions was approved by the National Malaria Board, a multi-sectorial advisory board within the Ministry of Health.

### Data sources and collection

The source of this study data is the national malaria surveillance database. It includes all subjects screened for malaria within the Surinamese health system. Access to diagnosis and treatment is free for all. This means that the surveillance data include subjects from the stable and mobile communities, including documented and undocumented migrants. Malaria diagnosis was done by microscopy screening of blood smears (parasite detection in 200 (routine screening) or 500 (non-routine screening) fields of a thick smear) or by RDTs. All RDT results were cross-checked with blood smears. As much as possible people with a positive diagnosis were provided with treatment on the spot.

A system for internal and external quality control of microscopy was in place. Slides (all positives, 10% of negatives) were sent to the national reference laboratory at the Bureau of Public Health (Ministry of Health) for re-check. The Bureau of Public Health took part in the regional External Quality Assurance Programme (EQAP). Microscopy refreshment training was organized on an annual basis (at least during the last decade).

**Fig. 1:**
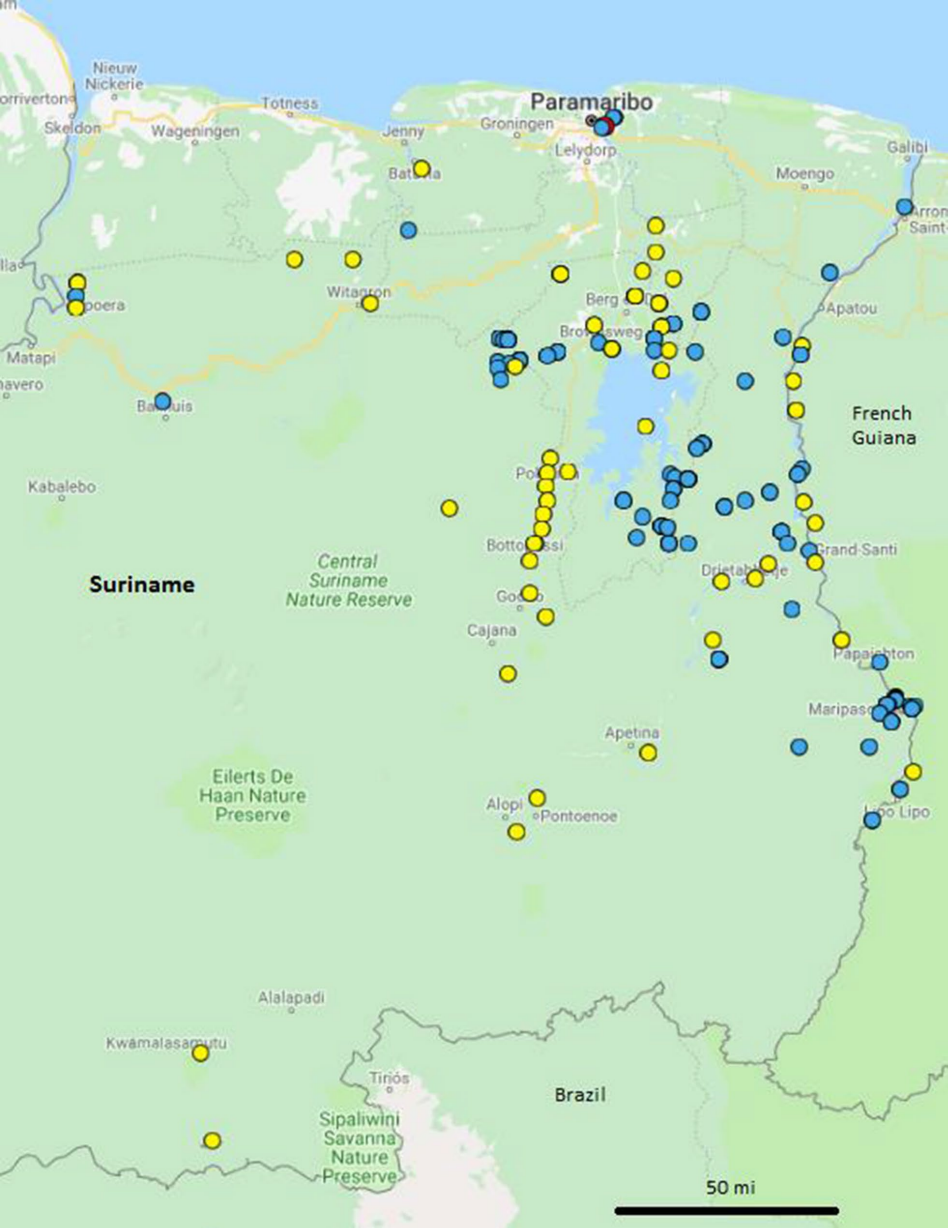
Geographical distribution of the Bureau of Public Health (red), the Medical Mission Primary Health Care clinics (yellow), and the Malaria Programme notification points (2007–2016; including ACD service areas, blue)

The Surinamese national surveillance system includes the following components: (i) the laboratory of the Bureau of Public Health reports on malaria data from the medical centre of the Bureau of Public Health, from hospitals, from Regional Health Services, from the blood bank and from private laboratories; (ii) the Medical Mission which has 56 clinics in the Interior (8); and, (iii) the Ministry of Health Malaria Programme (Malaria Programme), which targets its interventions at high-risk populations, especially mobile migrant gold miners. The programme has been supported throughout the years by the Global Fund to Fight AIDS, Tuberculosis and Malaria, the Inter-American Development Bank (IDB), the US Agency for International Development (USAID), the Pan American Health Organization and private companies (i.e., Newmont Mining Company). It provides malaria services in a migrant clinic in Paramaribo, operates a small number of border malaria-screening posts along the border with French Guiana, maintains a malaria service deliverer (MSD) network in gold-mining areas and performs ACD surveys in remote mining areas (Fig. 1).

The MSD network consists of lay people from the high-risk (mining) areas and communities (including migrants) who are trained and supervised by the Malaria Programme to provide health information, bed nets and diagnosis and treatment to their peers. Where possible a relationship has been established with local companies (mining and logging) to train MSDs among their personnel.

ACDs were almost exclusively done by the Malaria Programme MSDs and MSD-supervisors. Incidental ACDs were executed by the Medical Mission in the villages. During ACDs, mass screening for malaria was done with RDTs. Cross-checking of RDT results with blood smear took place during the ACDs in the field or after the ACDs at the central level.

National aggregation of malaria data in the surveillance system was done by the Malaria Programme. For the time-trend analysis, the data on subjects with a positive malaria test, diagnosed within the national health structures or identified during ACDs between 2000 and 2016, were used. Data on malaria-risk population, mortality and hospitalization as a result of malaria infection were maintained by the epidemiology unit of the Bureau of Public Health. These data were also used for the time-trend analysis.

To identify significant aggregation of cases in notification points over time or space, a spatiotemporal cluster-analysis was conducted. For this the screened subjects, both tested positive (cases) and negative (controls), registered between 2007 and 2016 by the Malaria Programme were used. This included passive and active screening.

For both analyses, malaria cases were defined as people in whom the, regardless of the presence or absence of clinical symptoms, presence of malaria parasites in the blood was confirmed by microscopic examination. In addition, people who in the absence of a blood smear result, had a positive RDT result were included as cases. Similarly, malaria-negative persons (controls) were defined as people with a negative blood smear result, or people who in the absence of a blood smear result, had a negative RDT result.

### Analysis and statistics

#### Morbidity and mortality trends

For the temporal trend analysis, graphs and trend lines were created using Epi Info™ version 7.2.1.0 (Centers for Disease Control and Prevention (CDC), Atlanta, GA, USA) and Tableau Software, version 9.2. The variables assessed over time were population at risk, number of malaria cases, malaria hospitalizations, and malaria deaths. For the persons screened, the variables gender and age (mean and standard deviation), country of origin, nationality, diagnosis (blood smear or RDT), and health service provider (organization) were assessed. For the positive cases, locality of infection (based on travel history; considering import versus autochthonous cases), and malaria parasite species were included.

Calculations were made for the Annual Parasite Index (API = total confirmed cases in a year × 1000/total population); the proportion of non-indigenous (imported) malaria cases (the total confirmed imported cases × 100/total confirmed cases); the proportional contribution of each parasite species to the total number of cases (total number of confirmed cases for one species × 100/total confirmed number of cases for all species); the proportion of cases reported/notified for each surveillance system (the number of cases per surveillance system × 100/total number of cases).

Trend lines on number of autochthonous malaria cases were evaluated with a linear trend model to determine significant changes over time (significance at p < 0.05). For the period 2014–2016 the autochthonous cases were mapped using Tableau software, version 9.2, in order to assess the presence of recent areas of transmission or so-called ‘hot spots’. For the purpose of mapping all national cases without known locality of infection were mapped in the capital of the country; Paramaribo and the Coastal Area of Suriname however have been malaria-free since 1968.

#### Spatiotemporal clusters of malaria cases by notification points

The space–time cluster analysis was conducted using SaTScan software (v.8.0) [4]. Specifically, a Bernoulli model was used to evaluate the distribution of positive malaria cases in notification points relative to the control group (which were defined as the suspected cases that were tested but were negative for malaria). The spatial and temporal unit of analysis was locality of notification and month, respectively. Notification points included the Malaria Programme clinic in Paramaribo, the border screening points (Albina, Tumatu, Antonio do Brinco) along the border with French Guiana and the MSD service points in the mining areas in the interior of Suriname. These notification points almost all exclusively provide malaria health services. For ACDs, the area of ACD was recorded as a notification point, with one coordinate for the survey.

A maximum spatial and temporal window of 50% of the study area and study period, respectively, was used as recommended by Kulldorf [19]. Significant clusters of high incidence (Bernoulli model, p < 0.05) were mapped using Google Earth Pro Version 7.3.0.3832 (32-bit) and cluster characteristics were described in a Table.

### Ethics approval

Ethics approval was received from the Committee for Human-centred Scientific Research (Ministry of Health) in Suriname (VG-15-17). Exception of ethical review was obtained from the Pan American Health Organization (PAHO) Ethical Review Committee considering that the study is based on routine programme information (PAHO-2017_07_066). The confidentiality of the study subjects was protected and individual data were not shared.

## Results

### Epidemiological trends

Between 2000 and 2016, a total of 139,667 people were seen for malaria by the national health services. Of these 71,793 (51.4%) were found positive and 62,179 were found negative for malaria by blood smear. In addition, 1622 malaria cases and 3094 negatives were identified in people without a blood smear result but with a RDT result. This resulted in a total of 73,415 confirmed malaria cases over the study period, of which 66,386 cases were autochthonous. Overall cross-checking of RDT results with blood smear was realized in 84.4% of the patients screened with RDTs. In 816 persons with a positive RDT result the blood smear was negative. Since microscopy result is the gold standard, these are considered false-positives, which excluded them from the malaria cases.

**Fig. 2:**
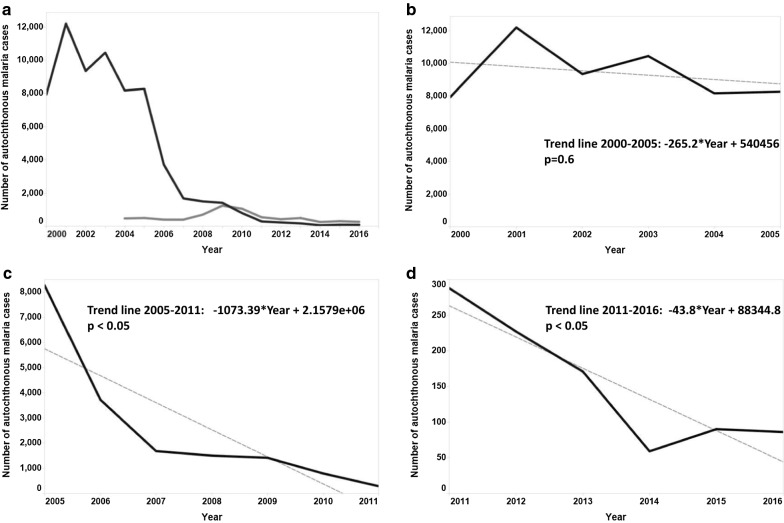
Number of autochthonous (black) and imported (grey) malaria cases in Suriname 2000–2016 (**a**) with a trend line for autochthonous cases over the periods 2000–2005 (**b**), 2005–2011 (**c**), and 2011–2016 (**d**). *Linear trend model; p < 0.05 is considered statistically significant

Malaria incidence, including both autochthonous and imported cases, in Suriname decreased significantly during the study period (– 95.6%), after an initial peak in 2001 of 12,197 cases to a low of 352 cases in 2016, of which 86 were autochthonous (Fig. 2a). After a dynamic period of high incidence between 2000 and 2005 with no significant decrease in number of malaria cases (Fig. 2b), a steep decrease was realized between 2005 and 2011 (p < 0.05; Fig. 2c). This was followed by a much more gradual decline since 2011 (p < 0.05, Fig. 2d). The API decreased from 167.5 in 2000, with a peak of 250.1 in 2001, to 1.0 in 2016 (Table 1).

**Table 1:** Annual Parasite Index, number of malaria hospitalizations and malaria deaths in Suriname (2000–2016)

Year	Annual Parasite Index	No. of malaria hospitalizations	No. of malaria deaths
2000	167.5	N.A.	24
2001	250.1	217	23
2002	185.2	323	16
2003	198.8	377	18
2004	149.3	163	7
2005	144.9	153	2
2006	62.9	50	0
2007	25.8	36	1
2008	23.1	51	0
2009	21.8	20	1
2010	9.6	13	1
2011	3.4	6	1
2012	2.7	10	0
2013	2.0	5	1
2014	0.7	6	0
2015	1.1	11	0
2016	1.0	12	0

As expected, the number of malaria hospitalizations and deaths, which included both national and imported cases, decreased with decreasing malaria incidence (Table 1). The last malaria-related death was recorded in 2013.

Between 2000 and 2003 all cases were reported by the Medical Mission. Since 2004 screening and reporting was also done by the Medical Centre of the Bureau of Public Health in Paramaribo. The Malaria Programme started reporting cases from 2006 onwards. Since then, due to changing risk populations, the contribution of the Malaria Programme to the national screening effort increased to 99.3% in 2016, which resulted in detection of 80.7% of the national number of cases for that year. In 2016, 60.0% of the people screened by the Malaria Programme were screened during ACDs.

**Fig. 3:**
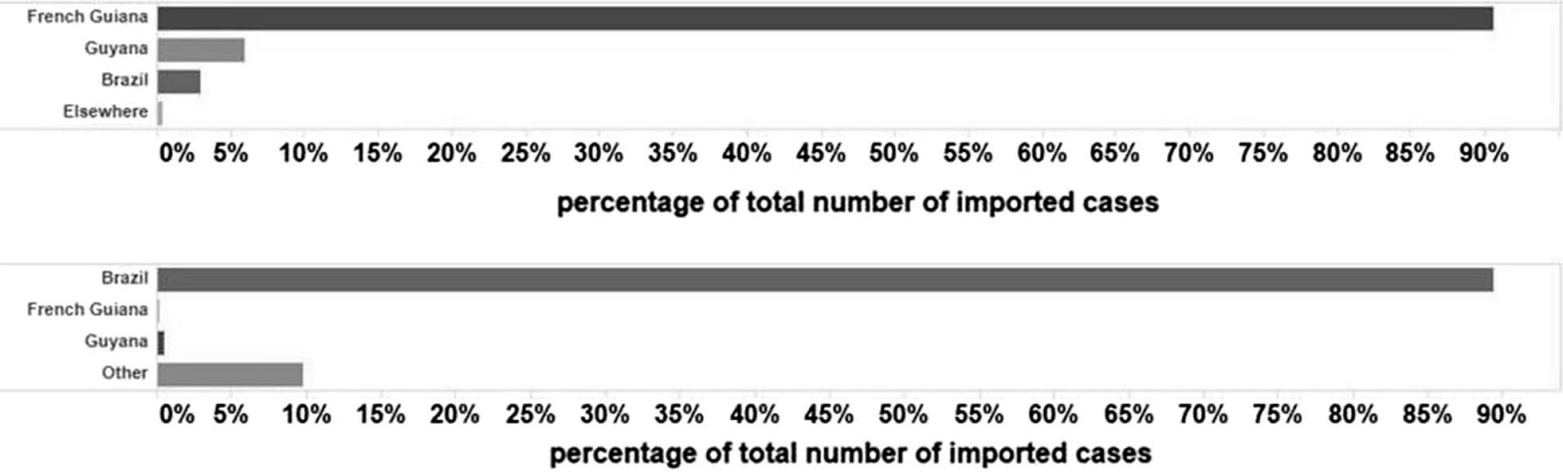
Contribution of countries (origin of imported cases (2004–2016) (**a**) and case nationalities (2007–2016) (**b**) to imported malaria cases in Suriname

Malaria imported cases were recorded separately since 2004. It steadily increased its proportion over time, from 5.4% (467 cases) of the total number of positives in 2004 to 75.6% (266 cases) in 2016. The majority of imported cases reported in Suriname originated from French Guiana (94.2% between 2004 and 2016; Fig. 3a) and possessed Brazilian nationality (89.4% between 2007 and 2016; Fig. 3b).

The median age (in years) of Surinamese malaria cases increased from 16 (0–90) in 2000 to 33 (6–54) in 2016. Neither autochthonous cases, < 5 years of age, nor pregnant autochthonous cases were recorded since 2013. The proportion of males showed an increasing trend among autochthonous malaria cases from 2004 (56.4%) to 2015 (78.9%) but went down again in 2016 (58.1%).

Similarly, the median age of imported cases increased from 25 (0–93) in 2004 to 33 (6–69) in 2016. Children < 5 years old were scarce among imported cases since 2010, with one child < 1 year in 2011 and one child of 4 years old in 2015. Three pregnant women were recorded among imported cases in 2016, the 2 years prior to that had no pregnant imported cases. In 2016, 62.0% of the imported cases were male.

**Fig. 4:**
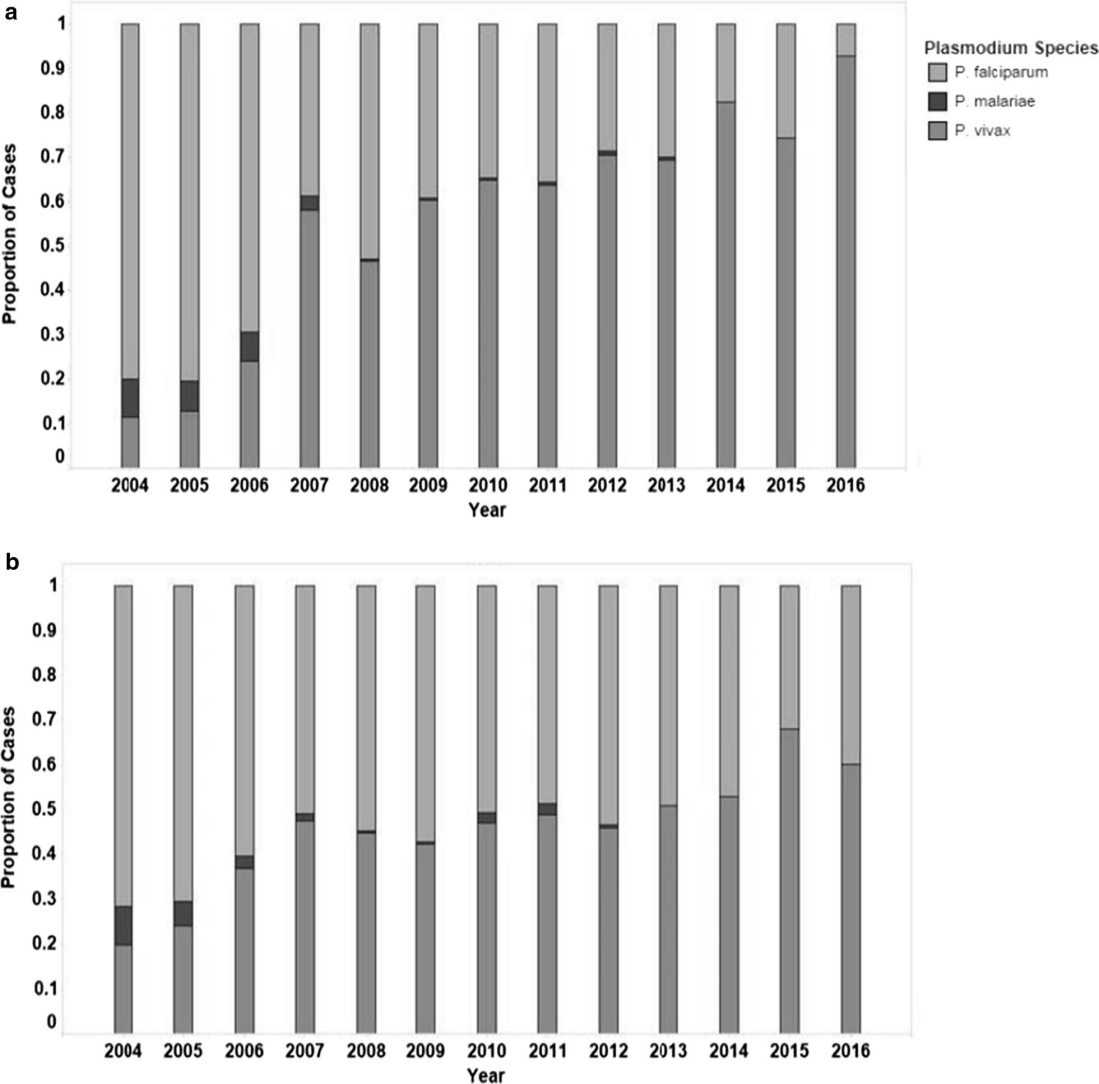
Proportion of cases per malaria parasite species for autochthonous (**a**) and imported cases (**b**) in Suriname (2004–2016)

*Plasmodium falciparum* was the predominant malaria species in Suriname until 2006, after which it declined to 7.1% (6 cases) of the autochthonous cases in 2016 (Fig. 4a). *Plasmodium falciparum* was still found in 39.9% (106 cases) of the imported cases in 2016 (Fig. 4b). Since 2007 *P. vivax* was the predominant species in Surinamese cases. *Plasmodium malariae* has not been seen in Suriname since 2013.

**Fig. 5:**
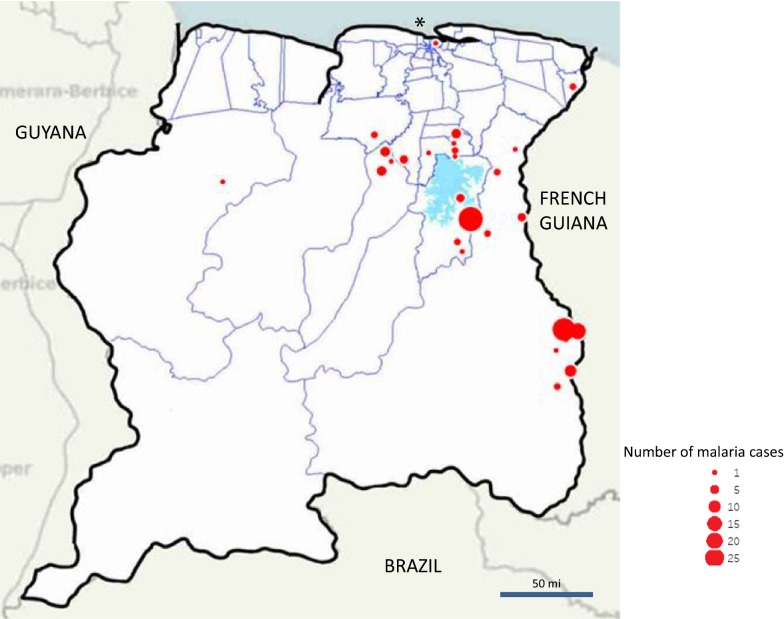
Map of autochthonous cases diagnosed in Suriname (2014–2016). *Cases without known locality of infection are mapped in the capital; Paramaribo however is malaria-free

The distribution of autochthonous cases from 2014 to 2016 (Fig. 5) shows that cases during the three last years of the study period were recorded especially in the south border with French Guiana and around the Brokopondo Lake. The localities of infection are in and immediately around the gold mines in those areas.

### Space–time cluster analysis

Between 2007 and 2016 the Malaria Programme notification points serviced a total of 73,839 people (78.4% non-Surinamese nationals) and reported 8804 malaria cases (96.1% non-Surinamese nationals) from a total of 87 localities in the area of service (including ACD localities). The 2007–2016 Malaria Programme database, after exclusion of records without diagnosis, records without date of diagnosis and records without geo-reference (total 1.6%), contained 72,660 subject records usable for space–time analysis, which included 8804 cases and 63,850 controls.

**Fig. 6:**
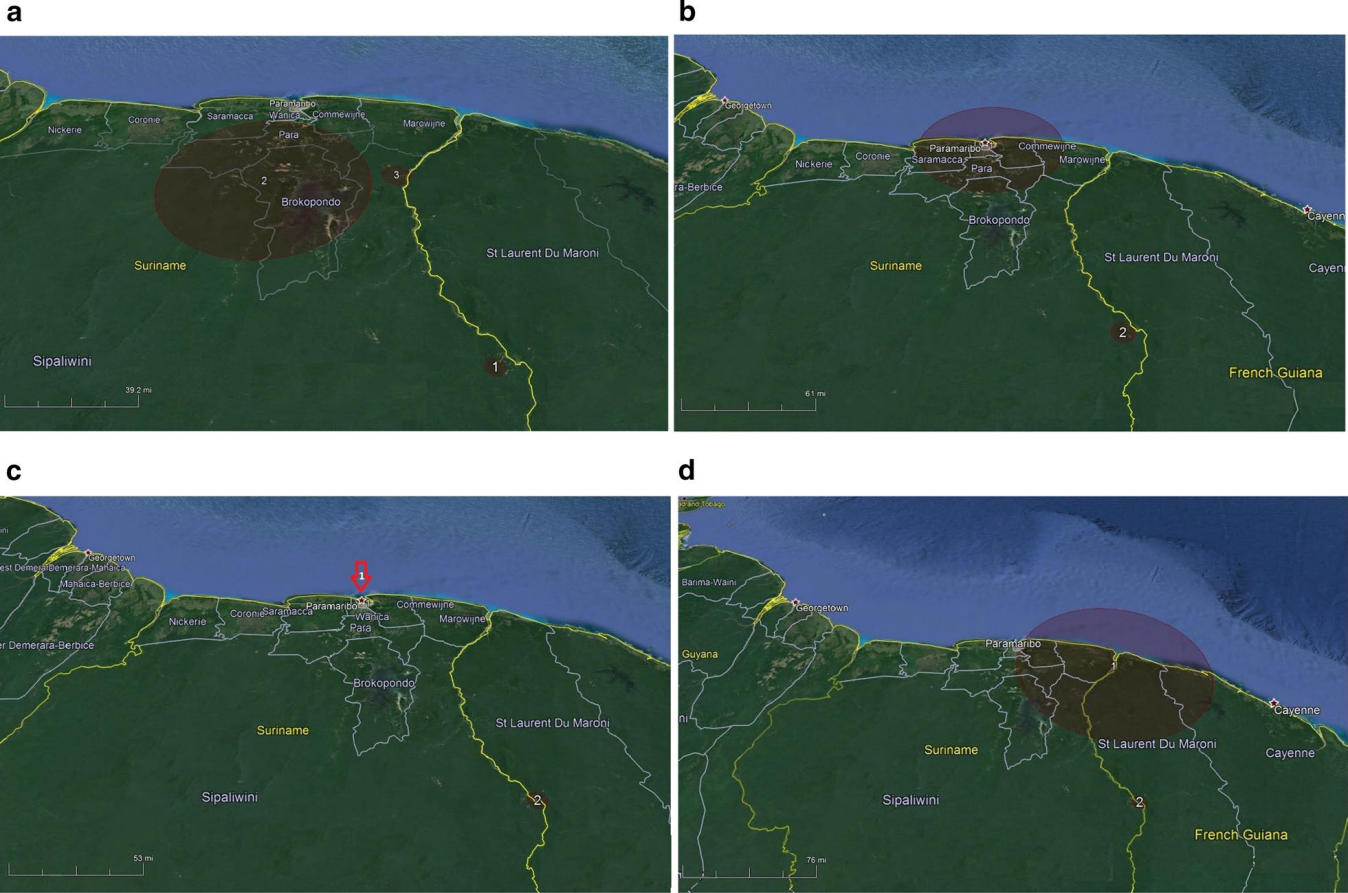
Space-time clusters of malaria cases identified within the Malaria Programme surveillance system in Suriname from 2007 to 2008 (**a**), 2009 to 2010 (**b**), 2011 to 2013 (**c**), and 2014 to 2016 (**d**) (for cluster data see Table 2)

For computational purposes, i.e., taking into account the processing capacity of the software, the space–time clustering was done in periods of 2 and 3 years as follows; 2007–2008 (2434 cases), 2009–2010 (3761 cases), 2011–2013 (1733 cases), and 2014–2016 (876 cases). Space–time cluster analysis of the Malaria Programme data 2007–2016 shows that in the 2007–2008 period three clusters can be defined, while the following three periods each have two clusters (Table 2, Fig. 6a–d). The notification points in cluster two in each period of analysis are located in one health service area along the south border with French Guiana. This area, the Lawatabiki area, has a radius of about 10 km and includes the notification points Benzdorp, Antino, Antonio do Brinco, Peruano, Vila Nova, and Cabanafo. The Malaria Programme clinic in Paramaribo, as a central level notification point, shows up as the first cluster in the periods 2, 3 and 4, while the Albina border screening point at the northern border with French Guiana becomes visible in the 2014–2016 cluster, right after its creation in 2015. The small cluster in the Brokopondo Lake area (Victoria, Krabudoin; 2007–2008, cluster 1) is mostly the result of ACDs and disappears in the more recent years. Likewise the cluster of Tumatu/Snesikondre (2007–2008, cluster 3) along the central part of the east border river is a very small and time constricted cluster that does not appear again. The percentage of positives in all cluster notification points is ranging from 9.1 to 100% during the cluster periods. The percentage of imported cases among the malaria cases increases over time, and is especially high in the more recent years.

**Table 2:** Space-time cluster analysis results of the Malaria Programme database from 2007 to 2016

Period of analysis	Identified cluster^a^	Cluster period (year/month)	Cluster diameter size (O/E)	Notification point	No. cases/No. control (% positive)	% Imported
2007–2008	1	2008/5–2008/12	75.3 km (1.8)	Krabudoin	4/4 (100%)	0
				Victoria	4/14 (28.6%)	0
	2	2008/8–2008/12	5.6 km (1.7)	Antino	201/311 (67.5%)	6.5
				Benzdorp	60/91 (65.9%)	0
	3	2008/10–2008/10	10.1 km (2.8)	Tumatu	2/2 (100%)	0
				Snesikondre	10/10 (100%)	0
2009–2010	1	2009/1–2009/12	79.6 km (1.8)	Paramaribo	1270/3086 (41.2%)	77.2
				Victoria	134/430 (31.2%)	0
	2	2009/1–2009/5	9.2 km (2.0)	Antino	62/190 (32.6%)	0
				Vila Nova	105/216 (48.6%)	32.4
				Benzdorp	112/223 (48.9%)	2.7
2011–2013	1	2011/1–2012/5	0.0 km (2.8)	Paramaribo	802/3208 (25.0%)	84.2
	2	2011/9–2013/2	7.6 km (5.1)	Antonio do Brinco	109/176 (61.9%)	99.1
				Cabanafo	2/8 (25.0%)	100
2014–2016	1	2015/7–2016/12	126.4 km (3.4)	Paramaribo	156/1240 (12.6%)	85.3
				Albina	120/1316 (9.1%)	95.8
	2	2014/10–2015/12	7.2 km (9.7)	Antonio do Brinco	52/94 (55.3%)	100
				Peruano	6/61 (9.8%)	100
				Cabanafo	45/99 (45.5%)	100

^a^ Only significant clusters (p < 0.05) are shown

## Discussion

The study shows that Suriname experienced a period of stable high malaria incidence from 2000 to 2005 (around 160 cases per 1000 habitants per year). This was followed by a 10-year period of a steep decline in malaria transmission, which for a large part, especially between 2005 and 2010, was the result of a decline in the stable village communities (8). Since 2011, Suriname has consistently reported low malaria parasite indexes (1.0 per 1000 habitants in 2006) and decreasing numbers of severe cases. Pregnant women, and children < 5 years old were no longer diagnosed with malaria. *Plasmodium malariae* disappeared and *P. falciparum* was almost eliminated. Over time mobile migrant gold miners became the priority risk populations.

The decline in malaria transmission in Suriname is clear progress towards malaria elimination. The success of malaria control in Suriname is thought to be linked to a number of interventions such as improved surveillance, prevention strategies and case management [7, 6]. Prevention strategies included the free distribution of long-lasting insecticide-impregnated bed nets to risk populations, combined with an intensive education and awareness building campaign. The mass distribution of nets actually took place in a time of climatic instability, the combination of which is thought to have severely impacted the mosquito population in known high malaria risk areas at that time [17].

Case management and surveillance were improved with the introduction of RDTs in 2003. ACDs were implemented in remote gold-mining areas, and border screening posts were created along the border with French Guiana. Furthermore, the so-called Malaria Service Deliverer (MSD) network, as an innovative approach proved successful in getting access to the new risk population, the mobile migrant populations in remote mining areas. It provided miners with low-threshold access to malaria services, and was instrumental in eliminating remaining remote malaria hot spots. The approach is currently being considered for the provision of other health services directed toward this specific, hard-to-reach, target population. The space–time analysis data show that with ACDs, the MSD network and the border screening posts clusters of high incidence were captured.

Case management was also improved with the change of *P. falciparum* treatment from quinine to ACT with a single dose of primaquine in 2004. In Suriname *P. falciparum* and *P. falciparum*/mixed infection were the cause of 89% of the total number of cases in 2004 [5]. ACT reduces gametocyte transmission to mosquitoes. Primaquine enforces this effect [20–22]. Similar to comparable situations in the region [3, 23], effective treatment of *P. falciparum* cases was followed by a steep decline in *P. falciparum* infections, resulting in *P. vivax* becoming relatively more important. Since the *P. falciparum* infections were mostly a problem for the Maroon populations along the Tapanahony River and the eastern border with French Guiana, the decline in Surinamese cases had a spill-over effect in French Guiana [24].

Malaria imported cases in Suriname experienced a relative increase over the same time period, from 5.4% (467 cases) of all notified cases in 2004, to 75.6% (266 cases in 2016. Imported cases are mainly Brazilian nationals travelling from French Guiana as the most probable point of infection. Clusters of cases by notification points varied over the time period (2007–2016); however, almost all clusters are located in the southern and northern border area between Suriname and French Guiana (Lawatabiki and Albina posts). Investing in border screening at known crossing-sites for miners was a sound decision to manage imported malaria. In addition there is clustering in the migrant clinic of the Malaria Programme in Paramaribo, which shows that it filled a need.

The Malaria Programme is currently responsible for the vast majority of the national screening effort and is intercepting both autochthonous and imported cases. The population serviced and cases identified in the central level clinic, the MSD network and during ACDs originate mostly from the migrant risk populations. The cluster analysis shows that the expansion of notification points at central level (migrant clinic) and along the Suriname-French Guiana border (MSD network) are important for targeting high-risk population and rapidly detecting imported malaria cases. The spatiotemporal analysis of the surveillance data provides insights into the involvement of cross-border moving populations in the Surinamese malaria transmission dynamics. It offers a case for continued investment in these critical components of the national malaria surveillance and management system.

The importance of the mobile cross-border moving populations and the role of miners in malaria transmission is recognized throughout the region [25, 26]. Malaria and other health challenges in migrant gold miners, however, are a largely unrecognized or neglected problem in French Guiana [27, 28]. As a result, the number of imported malaria cases from French Guiana in neighbouring countries far exceeds the number of French Guiana cases reported by French health authorities [24]. While in many countries the number of *P. falciparum* cases greatly reduced following the introduction of effective treatment, malaria in cases imported from French Guiana into Suriname is still mostly due to *P. falciparum* infections. This can likely be linked directly to the absence of diagnosis and treatment services in the illegal mining settings [29]. Not addressing this malaria reservoir in French Guiana raises the threat of drug resistance [30–32] and puts the population of the Guianas and Brazil at risk for new malaria outbreaks and re-introduction of malaria in areas where elimination has been achieved [17]. Monitoring of resistance to malaria treatment should be in place.

The present study provides a countrywide overview of the malaria situation in Suriname with robust data from both active and passive case detection. It is a first study to give insight into the spatial and temporal trends of the relative burden of autochthonous *versus* imported malaria cases. The use of geographical information systems and space–time geo-statistics is becoming increasingly important to guide interventions for malaria control programmes [33, 34, 13]. This analysis will contribute towards a better understanding of malaria dynamics in the country. It will also contribute to improve the multi-country regional collaboration to eliminate hot spots and to cost-effectively manage malaria cases among cross-border moving migrant populations.

There are several limitations in this study. Due to the presence of the Medical Mission in the stable communities and the provision of malaria services by the Malaria Programme along the border and in remote mining areas, free access to diagnosis and treatment is available in most areas. However, due to high travel costs and a low perception of health priority in the mining population, self-treatment is common [24, 35] and under-reporting will occur. This means that a part of malaria infections may be missed in the surveillance system. Particularly, it is known that passive case detection is subject to sub-notification due to limitations such as travelling costs [36]. Also the remoteness of the risk areas and the inherent characteristics of the MSD network (including a high turnover of MSDs) impact quality control and cross-checking of RDT results (despite continuous supervision and regular refreshment trainings) in that blood smears are sometime of insufficient quality or absent.

Reaching malaria elimination in Suriname in 2020 requires national ownership of the elimination goal and leadership in achieving this. It also requires the development of a strategy for migrant health care, which is currently not in place. In order to develop a migrant health strategy, further insights into the characteristics, size and health needs of this migrant population are needed. The MSD network provides a basis upon which to build better access to migrant populations. Integration of health services in the network may result in a more sustainable system of health provision to this population. In addition, actions could be taken to lower the threshold for migrants, as a result of language or other barriers, to other health service providers both in the interior and at the central level. For the management of the so-called ‘hidden reservoir’ of malaria in mobile migrant populations in French Guiana, regional involvement is indispensable. Some regional cooperation for malaria is already in place, specifically a tri-national, innovative, pilot project to self-diagnose and self-treat malaria among migrant gold miners active in French Guiana (Malakit) [37] but the results presented here indicate that a strong involvement and full commitment of all partners is required, as is being established elsewhere [4], especially in the light of recent increases in the number of malaria cases reported in the American Region, including Guyana, Venezuela and Brazil [38].

## Conclusion

Suriname has been successful in reducing malaria to near-elimination level. This success came about following a coordinated and adaptive (innovative) response by the Ministry of Health under guidance of the National Malaria Board, and with the support and funds of national and international partners. It could serve as a learning experience for other countries in the region. An important lesson learned is that bringing the interventions, especially bed nets and diagnosis and treatment, to the population at risk is a key element to achieve successful prevention and control. Surveillance data are an important tool to guide the programme and establish risk-based interventions. Innovative approaches can be developed to implement surveillance and case management in challenging situations, as was done with the MSD network in remote mining areas.

The national goal of malaria elimination by 2020 in Suriname will be hard to achieve if the number of malaria imported cases remains high. Re-introduction of malaria is a serious threat. A regional approach and collaboration within the Guianas and Brazil is essential. The elimination goal should be a regional goal.
